# Supplementary material for: A Likelihood Approach for Real-Time Calibration of Stochastic Compartmental Epidemic Models
Source: PLoS Comput Biol. 2017 Jan 17;13(1):e1005257. doi: 10.1371/journal.pcbi.1005257 (PMC5240920; doi:10.1371/journal.pcbi.1005257)
Supplement: S1 File — (TAR.GZ) [file pcbi.1005257.s014.tar.gz › HSPH_Online-SI-Revision/IPoi09/n100-extreme/n100-extreme_BENCH2_table8.pdf]

1	newly 3 weeks spec	655.	1127.92	{565., 2167.}	79.8598	0.203692	newly 3 weeks cumul	2650.	4794.89	{2370., 8858.}	86.0922	0.216188	newly 5 weeks spec	298.	583.639	{297., 1063.}	99.3289	0.237528	newly 5 weeks cumul	3391.	6187.03	{3048., 11 411.}	87.4572	0.218205	newly 46 weeks cumul	4195.	7683.85	{3790., 14 136.}	88.058	0.218509
2	newly 3 weeks spec	761.	697.639	{325., 1112.}	50.3199	0.230557	newly 3 weeks cumul	3077.	2789.08	{1283., 4424.}	49.2022	0.226766	newly 5 weeks spec	441.	401.738	{186., 637.}	50.7305	0.23163	newly 5 weeks cumul	4068.	3716.21	{1716., 5892.}	49.329	0.225536	newly 42 weeks cumul	5247.	4970.3	{2277., 7876.}	49.8717	0.220356
3	newly 3 weeks spec	277.	482.468	{229., 871.}	83.1781	0.207995	newly 3 weeks cumul	1321.	2161.58	{1007., 3863.}	75.2656	0.196672	newly 5 weeks spec	122.	230.855	{109., 413.}	96.0999	0.228137	newly 5 weeks cumul	1607.	2727.4	{1241., 4934.}	79.9115	0.204002	newly 47 weeks cumul	1853.	3248.94	{1445., 5919.}	84.2453	0.211016
4	newly 3 weeks spec	75.	195.492	{94., 369.}	161.705	0.345766	newly 3 weeks cumul	495.	1068.87	{518., 2042.}	117.006	0.269085	newly 5 weeks spec	26.	69.0984	{34., 115.}	166.267	0.356248	newly 5 weeks cumul	564.	1252.82	{615., 2401.}	122.933	0.280038	newly 51 weeks cumul	589.	1356.62	{667., 2609.}	130.788	0.294567
5	newly 3 weeks spec	383.	690.274	{307., 1243.}	88.9624	0.220728	newly 3 weeks cumul	1884.	3087.26	{1404., 5511.}	75.2791	0.197231	newly 5 weeks spec	164.	334.968	{141., 606.}	109.363	0.255113	newly 5 weeks cumul	2324.	3904.69	{1776., 7040.}	78.4861	0.202327	newly 48 weeks cumul	2679.	4657.08	{2088., 8438.}	83.048	0.209682
6	newly 3 weeks spec	620.	583.639	{297., 1063.}	47.34	0.209753	newly 3 weeks cumul	2591.	2520.07	{1245., 4720.}	46.9298	0.201152	newly 5 weeks spec	303.	302.213	{142., 579.}	47.3516	0.197875	newly 5 weeks cumul	3314.	3246.74	{1590., 6045.}	46.8598	0.199047	newly 46 weeks cumul	4005.	4016.89	{2034., 7445.}	47.0735	0.1953
7	newly 3 weeks spec	425.	1249.37	{549., 2262.}	193.97	0.382725	newly 3 weeks cumul	2093.	5067.5	{2240., 9098.}	142.866	0.307838	newly 5 weeks spec	238.	700.323	{308., 1261.}	194.687	0.379477	newly 5 weeks cumul	2658.	6706.58	{3002., 11 991.}	152.92	0.322911	newly 44 weeks cumul	3121.	8714.55	{3846., 15 629.}	179.435	0.362376
8	newly 3 weeks spec	611.	808.508	{383., 1490.}	54.9382	0.170754	newly 3 weeks cumul	2634.	3483.13	{1706., 6498.}	54.0044	0.167248	newly 5 weeks spec	315.	424.459	{209., 746.}	55.4307	0.167097	newly 5 weeks cumul	3371.	4491.23	{2202., 8307.}	54.5103	0.167258	newly 46 weeks cumul	4133.	5563.59	{2797., 10 286.}	55.4109	0.168362
9	newly 3 weeks spec	509.	1364.73	{647., 2526.}	168.119	0.354719	newly 3 weeks cumul	2442.	5674.	{2744., 10 283.}	132.76	0.300957	newly 5 weeks spec	256.	739.032	{351., 1319.}	188.684	0.380013	newly 5 weeks cumul	3053.	7427.18	{3573., 13 440.}	143.497	0.31754	newly 46 weeks cumul	3616.	9410.9	{4479., 16 956.}	160.257	0.343336
10	newly 3 weeks spec	32.	114.852	{56., 232.}	258.914	0.479665	newly 3 weeks cumul	165.	633.918	{321., 1274.}	284.193	0.512173	newly 5 weeks spec	7.	41.5246	{19., 84.}	493.208	0.697911	newly 5 weeks cumul	184.	744.541	{376., 1478.}	304.642	0.535552	newly 50 weeks cumul	192.	806.82	{406., 1602.}	320.219	0.552593
11	newly 3 weeks spec	582.	938.758	{433., 1646.}	75.0166	0.190436	newly 3 weeks cumul	2565.	3837.68	{1679., 6825.}	66.6528	0.180477	newly 5 weeks spec	318.	522.371	{232., 930.}	78.1954	0.195897	newly 5 weeks cumul	3307.	5060.37	{2198., 9016.}	69.2592	0.184143	newly 44 weeks cumul	4044.	6545.97	{2840., 11 724.}	75.753	0.193117
12	newly 3 weeks spec	488.	1000.62	{440., 1814.}	109.097	0.254784	newly 3 weeks cumul	2113.	4211.36	{1943., 7728.}	103.369	0.245659	newly 5 weeks spec	223.	526.197	{246., 917.}	138.095	0.304061	newly 5 weeks cumul	2630.	5465.8	{2544., 9902.}	110.928	0.258054	newly 45 weeks cumul	3083.	6842.23	{3115., 12 417.}	123.81	0.279193
13	newly 3 weeks spec	1177.	834.361	{400., 1433.}	54.0928	0.29844	newly 3 weeks cumul	4644.	3338.49	{1628., 5653.}	53.4411	0.291705	newly 5 weeks spec	679.	480.492	{234., 823.}	55.1462	0.304096	newly 5 weeks cumul	6187.	4450.97	{2160., 7519.}	53.7351	0.292971	newly 43 weeks cumul	8302.	5921.79	{2830., 10 165.}	54.4023	0.298659
14	newly 3 weeks spec	9.	495.607	{213., 773.}	5406.74	1.65784	newly 3 weeks cumul	64.	2352.34	{1041., 3588.}	3575.54	1.48484	newly 5 weeks spec	5.	218.672	{94., 360.}	4273.44	1.55437	newly 5 weeks cumul	74.	2905.98	{1291., 4440.}	3827.	1.51342	newly 48 weeks cumul	86.	3333.75	{1486., 5098.}	3776.46	1.50744
15	newly 3 weeks spec	73.	328.629	{172., 525.}	350.177	0.59084	newly 3 weeks cumul	382.	1583.21	{823., 2573.}	314.453	0.554656	newly 5 weeks spec	19.	143.71	{79., 252.}	656.367	0.811632	newly 5 weeks cumul	449.	1947.55	{1011., 3152.}	333.752	0.574354	newly 48 weeks cumul	476.	2226.94	{1120., 3573.}	367.844	0.60749
16	newly 3 weeks spec	111.	334.967	{154., 519.}	201.772	0.399416	newly 3 weeks cumul	653.	1569.85	{679., 2431.}	141.325	0.303876	newly 5 weeks spec	31.	144.049	{63., 225.}	364.675	0.584604	newly 5 weeks cumul	748.	1932.57	{836., 2989.}	158.659	0.332011	newly 48 weeks cumul	812.	2216.3	{965., 3422.}	173.032	0.354422
17	newly 3 weeks spec	848.	1414.28	{694., 2424.}	76.7186	0.194217	newly 3 weeks cumul	3379.	5626.92	{2835., 9699.}	74.8466	0.19188	newly 5 weeks spec	459.	834.361	{400., 1433.}	88.5067	0.211442	newly 5 weeks cumul	4480.	7551.13	{3763., 12 928.}	76.8454	0.194494	newly 43 weeks cumul	5685.	10 134.4	{4942., 17 440.}	84.6683	0.206126
18	newly 3 weeks spec	371.	1416.77	{639., 2412.}	281.88	0.501518	newly 3 weeks cumul	1789.	5701.52	{2585., 9784.}	218.698	0.429457	newly 5 weeks spec	168.	811.306	{337., 1373.}	382.921	0.597341	newly 5 weeks cumul	2207.	7591.23	{3379., 12 945.}	243.961	0.460103	newly 44 weeks cumul	2601.	10 010.1	{4386., 17 088.}	284.857	0.505582
19	newly 3 weeks spec	522.	1127.92	{565., 2167.}	117.772	0.272313	newly 3 weeks cumul	2300.	4794.89	{2370., 8858.}	110.509	0.262042	newly 5 weeks spec	225.	583.639	{297., 1063.}	160.08	0.34507	newly 5 weeks cumul	2858.	6187.03	{3048., 11 411.}	118.044	0.275239	newly 46 weeks cumul	3344.	7683.85	{3790., 14 136.}	130.71	0.297197
20	newly 3 weeks spec	31.	195.492	{94., 369.}	530.619	0.724411	newly 3 weeks cumul	168.	1068.87	{518., 2042.}	536.231	0.733428	newly 5 weeks spec	4.	69.0984	{34., 115.}	1627.46	1.16686	newly 5 weeks cumul	185.	1252.82	{615., 2401.}	577.2	0.760465	newly 51 weeks cumul	187.	1356.62	{667., 2609.}	625.467	0.79077
21	newly 3 weeks spec	203.	495.607	{213., 773.}	144.916	0.308082	newly 3 weeks cumul	1027.	2352.34	{1041., 3588.}	131.052	0.288777	newly 5 weeks spec	76.	218.672	{94., 360.}	188.287	0.375137	newly 5 weeks cumul	1233.	2905.98	{1291., 4440.}	137.194	0.298669	newly 48 weeks cumul	1341.	3333.75	{1486., 5098.}	149.355	0.317937
22	newly 3 weeks spec	138.	324.1	{166., 487.}	135.7	0.307178	newly 3 weeks cumul	788.	1620.73	{853., 2365.}	108.549	0.26185	newly 5 weeks spec	76.	133.2	{68., 184.}	81.6667	0.210904	newly 5 weeks cumul	953.	1957.82	{1017., 2828.}	108.379	0.261849	newly 49 weeks cumul	1046.	2183.8	{1127., 3166.}	111.396	0.266264
23	newly 3 weeks spec	646.	1182.23	{539., 1929.}	89.7478	0.218307	newly 3 weeks cumul	2717.	4695.72	{2209., 7548.}	80.8836	0.205782	newly 5 weeks spec	343.	697.639	{325., 1112.}	107.724	0.246911	newly 5 weeks cumul	3563.	6302.57	{2978., 10 043.}	84.3487	0.210522	newly 42 weeks cumul	4442.	8483.77	{3970., 13 494.}	96.1581	0.229621
24	newly 3 weeks spec	888.	1292.89	{475., 2416.}	73.9636	0.200934	newly 3 weeks cumul	3507.	4932.14	{1955., 9152.}	67.8193	0.19564	newly 5 weeks spec	441.	795.953	{282., 1399.}	99.9539	0.218311	newly 5 weeks cumul	4589.	6756.39	{2597., 12 421.}	73.142	0.199116	newly 42 weeks cumul	5895.	9369.89	{3532., 16 672.}	82.3646	0.204671
25	newly 3 weeks spec	135.	1000.62	{440., 1814.}	641.202	0.793417	newly 3 weeks cumul	801.	4211.36	{1943., 7728.}	425.763	0.647298	newly 5 weeks spec	46.	526.197	{246., 917.}	1043.91	0.979158	newly 5 weeks cumul	936.	5465.8	{2544., 9902.}	483.953	0.69184	newly 45 weeks cumul	1018.	6842.23	{3115., 12 417.}	572.125	0.751478
26	newly 3 weeks spec	1048.	1089.85	{531., 1796.}	49.0661	0.192238	newly 3 weeks cumul	4173.	4345.02	{2143., 7404.}	47.5449	0.186448	newly 5 weeks spec	644.	631.984	{302., 1043.}	49.6563	0.206548	newly 5 weeks cumul	5651.	5811.36	{2847., 9880.}	47.9259	0.19023	newly 43 weeks cumul	7533.	7762.67	{3751., 13 349.}	48.756	0.193076
27	newly 3 weeks spec	806.	700.323	{308., 1261.}	53.2738	0.25422	newly 3 weeks cumul	3332.	2888.45	{1279., 5155.}	52.5772	0.250274	newly 5 weeks spec	451.	382.694	{165., 697.}	54.7422	0.266897	newly 5 weeks cumul	4401.	3793.52	{1668., 6779.}	53.0107	0.253629	newly 44 weeks cumul	5811.	4896.42	{2134., 8793.}	53.7168	0.262147
28	newly 3 weeks spec	568.	317.79	{162., 643.}	56.267	0.361728	newly 3 weeks cumul	2482.	1476.06	{791., 2864.}	54.6256	0.33745	newly 5 weeks spec	266.	138.903	{70., 270.}	57.6401	0.387597	newly 5 weeks cumul	3132.	1826.97	{962., 3545.}	55.1899	0.345191	newly 48 weeks cumul	3853.	2111.23	{1125., 4034.}	56.4018	0.365586
29	newly 3 weeks spec	769.	1541.36	{732., 2438.}	103.728	0.245166	newly 3 weeks cumul	3241.	6033.41	{2916., 9583.}	90.9186	0.226126	newly 5 weeks spec	412.	909.213	{417., 1383.}	122.155	0.270863	newly 5 weeks cumul	4213.	8124.85	{3878., 12 895.}	96.926	0.235133	newly 42 weeks cumul	5339.	11 003.7	{5193., 17 458.}	108.975	0.254547
30	newly 3 weeks spec	278.	114.852	{56., 232.}	63.6985	0.476644	newly 3 weeks cumul	1314.	633.918	{321., 1274.}	58.7981	0.411941	newly 5 weeks spec	87.	41.5246	{19., 84.}	58.5265	0.417906	newly 5 weeks cumul	1559.	744.541	{376., 1478.}	59.0017	0.414765	newly 51 weeks cumul	1722.	806.82	{406., 1602.}	59.5933	0.421575
31	newly 3 weeks spec	58.	80.3387	{41., 113.}	54.3103	0.155111	newly 3 weeks cumul	360.	487.387	{256., 701.}	51.353	0.149142	newly 5 weeks spec	22.	24.2097	{11., 38.}	47.5806	0.178897	newly 5 weeks cumul	413.	556.532	{295., 797.}	50.8279	0.14763	newly 52 weeks cumul	442.	587.226	{310., 842.}	50.0803	0.147555
32	newly 3 weeks spec	101.	286.258	{144., 472.}	183.424	0.386099	newly 3 weeks cumul	549.	1426.31	{724., 2259.}	159.801	0.350571	newly 5 weeks spec	30.	119.597	{59., 182.}	298.656	0.532594	newly 5 weeks cumul	633.	1727.47	{889., 2730.}	172.902	0.371637	newly 49 weeks cumul	682.	1936.55	{996., 3083.}	183.951	0.388911
33	newly 3 weeks spec	918.	623.226	{287., 1066.}	56.42	0.315963	newly 3 weeks cumul	3832.	2727.76	{1275., 4920.}	54.712	0.295853	newly 5 weeks spec	479.	305.323	{128., 585.}	59.2902	0.348309	newly 5 weeks cumul	4959.	3463.58	{1600., 6305.}	55.5588	0.304798	newly 47 weeks cumul	6261.	4184.74	{1922., 7627.}	56.7754	0.32101
34	newly 3 weeks spec	843.	1182.23	{539., 1929.}	60.8891	0.177958	newly 3 weeks cumul	3518.	4695.72	{2209., 7548.}	56.3859	0.174561	newly 5 weeks spec	471.	697.639	{325., 1112.}	66.4544	0.184812	newly 5 weeks cumul	4638.	6302.57	{2978., 10 043.}	58.0504	0.17593	newly 42 weeks cumul	6028.	8483.77	{3970., 13 494.}	61.0452	0.17847
35	newly 3 weeks spec	693.	623.226	{287., 1066.}	51.0962	0.227476	newly 3 weeks cumul	3121.	2727.76	{1275., 4920.}	50.4816	0.230713	newly 5 weeks spec	338.	305.323	{128., 585.}	53.6171	0.238697	newly 5 weeks cumul	3977.	3463.58	{1600., 6305.}	51.0962	0.234437	newly 47 weeks cumul	4760.	4184.74	{1922., 7627.}	51.2429	0.232856
36	newly 3 weeks spec	635.	396.145	{184., 759.}	56.3246	0.340079	newly 3 weeks cumul	2904.	1674.77	{805., 3038.}	57.137	0.360129	newly 5 weeks spec	364.	209.935	{89., 340.}	58.4899	0.371787	newly 5 weeks cumul	3816.	2172.21	{1018., 3901.}	57.6752	0.36662	newly 45 weeks cumul	4844.	2722.76	{1271., 4835.}	57.8804	0.370763
37	newly 3 weeks spec	121.	225.754	{115., 431.}	90.9362	0.232753	newly 3 weeks cumul	634.	1171.64	{602., 2288.}	87.9557	0.224347	newly 5 weeks spec	49.	83.9016	{42., 161.}	79.1235	0.207458	newly 5 weeks cumul	759.	1392.67	{720., 2726.}	86.7103	0.221529	newly 50 weeks cumul	824.	1524.85	{793., 2990.}	87.9397	0.223689
38	newly 3 weeks spec	12.	225.754	{115., 431.}	1781.28	1.21491	newly 3 weeks cumul	113.	1171.64	{602., 2288.}	936.849	0.958309	newly 5 weeks spec	3.	83.9016	{42., 161.}	2696.72	1.38006	newly 5 weeks cumul	121.	1392.67	{720., 2726.}	1050.97	1.00359	newly 50 weeks cumul	126.	1524.85	{793., 2990.}	1110.2	1.02548
39	newly 3 weeks spec	302.	1249.37	{549., 2262.}	313.699	0.531107	newly 3 weeks cumul	1471.	5067.5	{2240., 9098.}	244.494	0.45752	newly 5 weeks spec	115.	700.323	{308., 1261.}	508.976	0.693333	newly 5 weeks cumul	1796.	6706.58	{3002., 11 991.}	273.418	0.490364	newly 44 weeks cumul	2002.	8714.55	{3846., 15 629.}	335.292	0.554254
40	newly 3 weeks spec	529.	583.639	{297., 1063.}	48.6566	0.182551	newly 3 weeks cumul	2325.	2520.07	{1245., 4720.}	47.6425	0.182091	newly 5 weeks spec	275.	302.213	{142., 579.}	48.3696	0.182221	newly 5 weeks cumul	2963.	3246.74	{1590., 6045.}	47.7628	0.180067	newly 45 weeks cumul	3697.	4016.89	{2034., 7445.}	47.8727	0.182315
41	newly 3 weeks spec	253.	623.226	{287., 1066.}	146.793	0.308894	newly 3 weeks cumul	1228.	2727.76	{1275., 4920.}	123.092	0.269785	newly 5 weeks spec	114.	305.323	{128., 585.}	168.393	0.338326	newly 5 weeks cumul	1511.	3463.58	{1600., 6305.}	130.074	0.281135	newly 46 weeks cumul	1685.	4184.74	{1922., 7627.}	148.686	0.312567
42	newly 3 weeks spec	23.	137.131	{71., 265.}	496.222	0.717862	newly 3 weeks cumul	166.	722.623	{384., 1387.}	335.315	0.581731	newly 5 weeks spec	8.	50.2295	{25., 88.}	527.869	0.735092	newly 5 weeks cumul	186.	856.754	{446., 1670.}	360.62	0.605709	newly 50 weeks cumul	187.	938.705	{493., 1818.}	401.981	0.643219
43	newly 3 weeks spec	681.	1414.28	{694., 2424.}	110.074	0.251604	newly 3 weeks cumul	3118.	5626.92	{2835., 9699.}	85.3889	0.209778	newly 5 weeks spec	377.	834.361	{400., 1433.}	122.994	0.271829	newly 5 weeks cumul	4043.	7551.13	{3763., 12 928.}	91.0304	0.218828	newly 43 weeks cumul	5018.	10 134.4	{4942., 17 440.}	104.567	0.241477
44	newly 3 weeks spec	174.	181.565	{95., 289.}	44.5032	0.172839	newly 3 weeks cumul	824.	916.903	{470., 1461.}	43.979	0.158396	newly 5 weeks spec	61.	75.0484	{38., 123.}	48.8895	0.160078	newly 5 weeks cumul	988.	1111.55	{577., 1762.}	44.1687	0.157052	newly 49 weeks cumul	1084.	1245.58	{646., 1996.}	44.5393	0.154505
45	newly 3 weeks spec	45.	328.629	{172., 525.}	630.287	0.800951	newly 3 weeks cumul	244.	1583.21	{823., 2573.}	548.856	0.749329	newly 5 weeks spec	9.	143.71	{79., 252.}	1496.77	1.13614	newly 5 weeks cumul	276.	1947.55	{1011., 3152.}	605.633	0.785691	newly 48 weeks cumul	280.	2226.94	{1120., 3573.}	695.334	0.837939
46	newly 3 weeks spec	223.	988.839	{463., 1749.}	343.425	0.570936	newly 3 weeks cumul	1040.	4387.35	{2028., 7877.}	321.861	0.552288	newly 5 weeks spec	61.	482.468	{229., 871.}	690.931	0.819583	newly 5 weeks cumul	1223.	5560.1	{2572., 9991.}	354.628	0.583672	newly 48 weeks cumul	1322.	6647.45	{3010., 12 047.}	402.833	0.626596
47	newly 3 weeks spec	132.	225.754	{115., 431.}	77.9309	0.208116	newly 3 weeks cumul	614.	1171.64	{602., 2288.}	93.0475	0.233865	newly 5 weeks spec	43.	83.9016	{42., 161.}	99.085	0.243835	newly 5 weeks cumul	740.	1392.67	{720., 2726.}	90.6712	0.228968	newly 50 weeks cumul	804.	1524.85	{793., 2990.}	91.893	0.231265
48	newly 3 weeks spec	186.	364.55	{165., 557.}	99.6505	0.248791	newly 3 weeks cumul	969.	1857.67	{868., 2782.}	95.6106	0.240901	newly 5 weeks spec	49.	140.083	{66., 215.}	187.721	0.401607	newly 5 weeks cumul	1115.	2220.55	{1022., 3319.}	102.384	0.25355	newly 50 weeks cumul	1182.	2456.42	{1109., 3660.}	110.137	0.267254
49	newly 3 weeks spec	8.	40.	{20., 64.}	400.403	0.627631	newly 3 weeks cumul	68.	251.597	{123., 349.}	269.995	0.504402	newly 5 weeks spec	3.	11.2097	{4., 20.}	274.731	0.486258	newly 5 weeks cumul	72.	283.919	{143., 395.}	294.332	0.53244	newly 52 weeks cumul	76.	296.113	{152., 418.}	289.622	0.527846
50	newly 3 weeks spec	733.	1414.28	{694., 2424.}	96.8041	0.226974	newly 3 weeks cumul	2944.	5626.92	{2835., 9699.}	94.3414	0.226267	newly 5 weeks spec	408.	834.361	{400., 1433.}	107.385	0.243507	newly 5 weeks cumul	3889.	7551.13	{3763., 12 928.}	97.338	0.230343	newly 43 weeks cumul	4985.	10 134.4	{4942., 17 440.}	105.747	0.243591
